# A structured curriculum for the acquisition of basic surgical endoscopic skills for surgical residents and quantification of skills improvement

**DOI:** 10.1016/j.sopen.2024.05.021

**Published:** 2024-06-08

**Authors:** Marc Fischer, Michail Galanis, Konstantinos Gioutsos, Jon Andri Lutz, Filipe Azenha Figueiredo, Patrick Dorn

**Affiliations:** Department of Thoracic Surgery, Inselspital, University Hospital of Bern, 3010 Bern, Switzerland

**Keywords:** Simulator training, Endoscopic skills, Surgical curriculum

## Abstract

**Introduction:**

New strategies and methods are needed to ensure that new generations can train and acquire surgical skills in a safe environment.

**Materials and methods:**

From January 2020 to October 2020, we performed a single centre, prospective observational cohort study. 19 participants (15 students, 4 residents) enrolled and 16 participants (13 students, 3 residents) successfully completed the curriculum. We performed a quantitative data analysis to evaluate its effectiveness in gaining and improving basic surgical endoscopic skills.

**Results:**

The time for single knot tying pre-, mid-, and post-training was reduced significantly, the average time (*sec*) decreased by 79.5 % (*p* < 0.001), the total linear distance (cm) by 74.5 % (*p* < 0.001) and the total angular distance (rad) by 71.7 % (p < 0.001). The average acceleration (mm/s^2^) increased by 20 % (*p* = 0.041). Additionally, the average speed increased by 23.5 % (*p* < 0.001), while motion smoothness (m/s^3^) increased by 20.4 % (*p* = 0.02).

**Conclusion:**

The obtained performance scores showed a significant increase in participants improving their basic surgical performance skills on the endoscopic simulator. This curriculum can be easily implemented in any surgical specialty as part of the residency training curriculum before first exposure in the operation room. All 16 participants recommended the implementation of such simulator training in their surgical training curriculum.

## Highlights

• Curriculum that can be easily adapted in surgical clinics
• 4 exercises, 10 h of training
• Gaining surgical endoscopic skills while reducing time and optimizing speed, accuracy and smoothness
• A structured surgical curriculum is of a crucial meaning
• Prior surgical experience or simulation exposure is not a prerequisite for entry into such a curriculum.

## Introduction

Enduring the journey to become a certified surgeon is a tremendous task for young, aspiring doctors. In addition to the demanding daily clinical routine, surgical procedures are becoming increasingly complex and require surgical, technical and endoscopic skills applied with the latest generation of equipment developed by industry partners to achieve the best outcome for surgical patients. The modern surgical working environment requires a higher level of administrative activity. At the same time, training procedures in the operation room and participation in training programs are decreasing due to the lack of time during statutory working hours [1,2]. Therefore, an efficient and performance-oriented way of teaching and training is required.

According to the Swiss Medical Federation (FMH), the average training time to complete a surgical specialty is 8.13 years, taking into consideration surgical specialties with a strong focus on endoscopic procedures for the period between 2012 and 2021. This statistic includes physicians who were younger than 26 years at the time of licensure and received their first board certification between 5 and 10 years, depending on the surgical specialty (i.e. otorhinolaryngology (ENT) 5 years, general surgery 6 years etc.). Residents in the following surgical specialties were included in the calculation: General Surgery, Gynecology and Obstetrics, Cardiovascular and Thoracic Surgery, Urology, ENT, and Pediatric Surgery, as these specialties require endoscopic surgical skills as described in the respective curriculum. The situation does not look better for any single specialty. According to the existing data, there is not a single surgical specialty in which endoscopic procedures are frequently performed by residents during the minimum training period (e.g., six years for general surgery and most surgical specialties) [3] (Table 1).

**Table 1:**
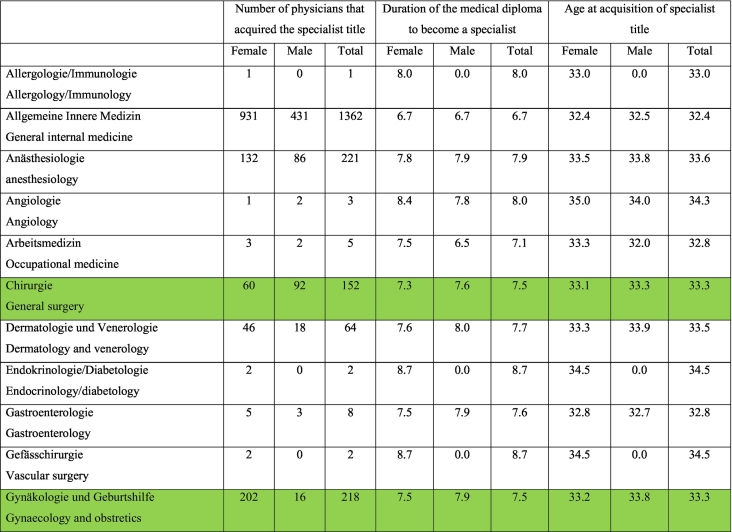

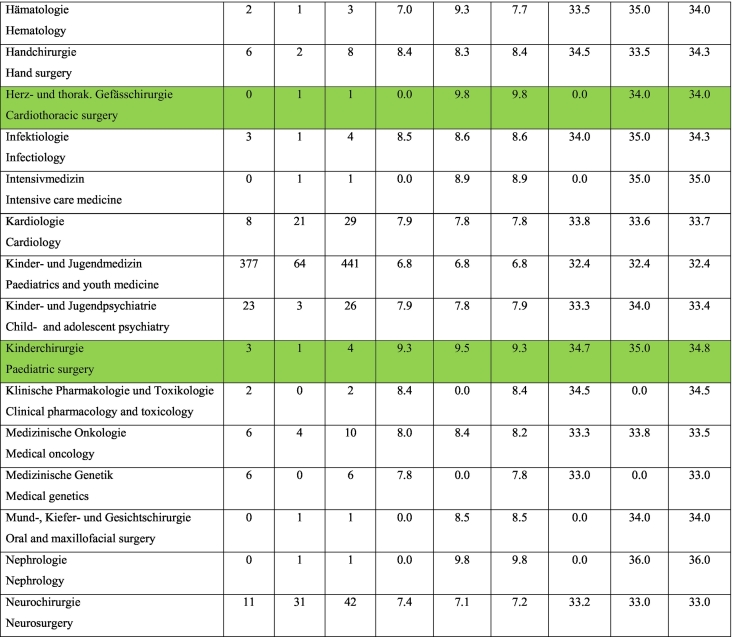

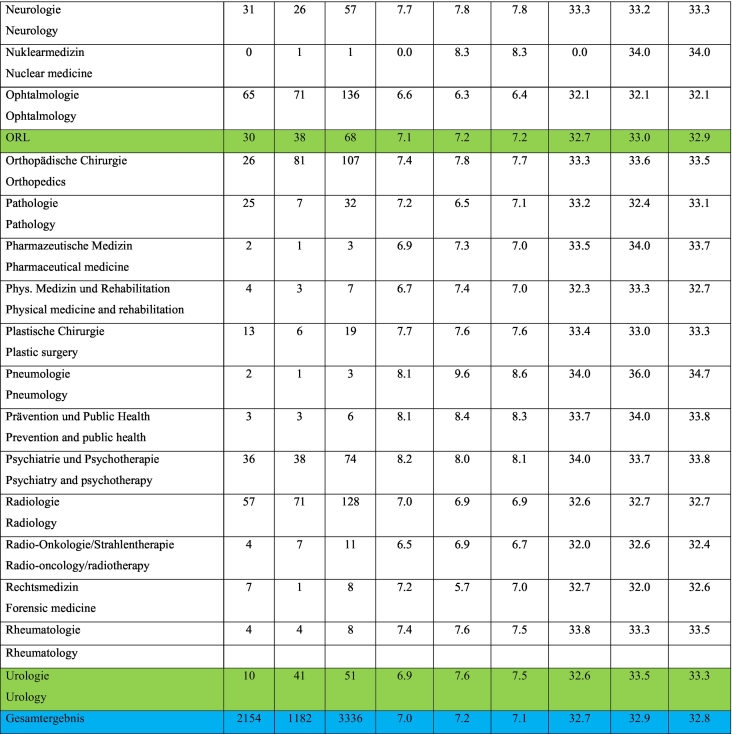
24.01.2023 FMH physician statistics 2012–2022. Doctors who have obtained a federal specialist title, years 2012–2022. Inclusion criteria: only federally qualified doctors, 1st specialist title and age ≤ 26 years when obtaining CH medical diploma, duration of WB > 5 and <10 years (*n* = 3336).

The new generation of surgical residents, not only in Switzerland but worldwide, is confronted with this reality. Due to concerns about patient safety and the aforementioned complexity of modern surgical approaches of novel interventions, new ways of training residents are emerging [1,2].

Furthermore, the traditional Halsted approach of “see one, do one, teach one” is increasingly being considered. On the other hand, structured surgical curricula based on a competency-based paradigm are being proposed.

The same point of view is taken by the Institute for Postgraduate Education in Switzerland, which wants to develop specialist programs with entrusted professional activities (EPA) into competency-based programs [5]. Therefore, simulator training curricula can be a valuable part of the solution for training surgical trainees and help to overcome some of the underlying challenges. According to Papanikolaou et al., simulator training for surgical trainees increases safety, improves efficiency, and can reduce costs [2]. However, the impact of surgical simulator training in a surgical curriculum still needs to be better understood.

At the Department of Thoracic Surgery, Inselspital, University Hospital of Bern, Bern, Switzerland, we contribute to research by organizing a two-day endoscopic workshop on a surgical simulator to find out if and how the learning curve for basic surgical skills in minimally invasive surgery changes through a thorough training program using measured quantitative data [6].

## Materials and methods

Between January 2020 and October 2020, we conducted a prospective observational cohort study in the Department of Thoracic Surgery at Inselspital Bern. 19 participants (15 students, 4 residents) were recruited in the first instance. The recruitment process for this study was meticulously conducted at the Department of Thoracic Clinic at the University Hospital Bern and through social media. In addition, residents and students who rotated in the department for thoracic surgery were approached, ensuring a diverse and comprehensive participant pool. This process continued until the required number of participants was fulfilled, further enhancing the validity of our findings.

The program was meticulously overseen by 3 surgeons of the staff team, who not only served as observers but also as administrators. Their dual roles ensured the integrity and thoroughness of the study, adding to its credibility.

### Ethic committee approval

All 16 participants who had successfully completed the curriculum (13 Students, 3 residents) gave their consent to participate in the planned study and the further analysis of their data. They were informed that they could discontinue their participation in the study at any time and withdraw their consent. Finally, their data was used in anonymized form for further statistical analysis. An ethics committee approval was not required due to the nature of the study.

### The curriculum

**Fig. 1:**
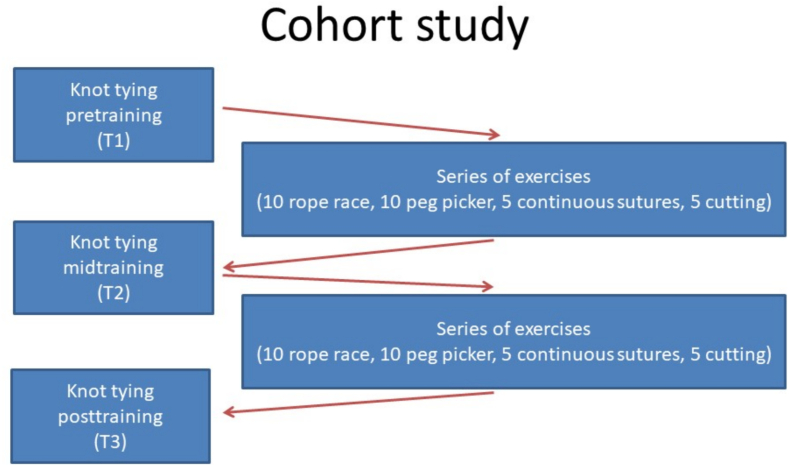
Sequence of the study.

The training curriculum consisted of 2 training rounds with 4 exercises (Fig. 1). Progress was documented by repeating the specific task of tying an endoscopic knot at the beginning (T1), at the middle (T2), and at the end of the training (T3). Before starting the initial round of training, the first knot-tying exercise test (T1) was performed. After the first round of 4 exercises and 30 repeats, the next knot-tying exercise test (T2) was performed. A second round of the same training exercises was performed and the training was completed with the final knot tie test (T3). Each participant invested between 12 and 20 h in the training program, spread over 2 days.

Each participant was individually familiarized with the simulator and the software navigation. Subsequently, the function of the individual surgical instruments was then demonstrated. In addition, an instructional video was shown before each task, explaining the steps necessary to obtain the required basic surgical skills. The participants were able to watch this video as many times as necessary during each phase of the training before each exercise [6].

### The training rounds

**Fig. 2:**
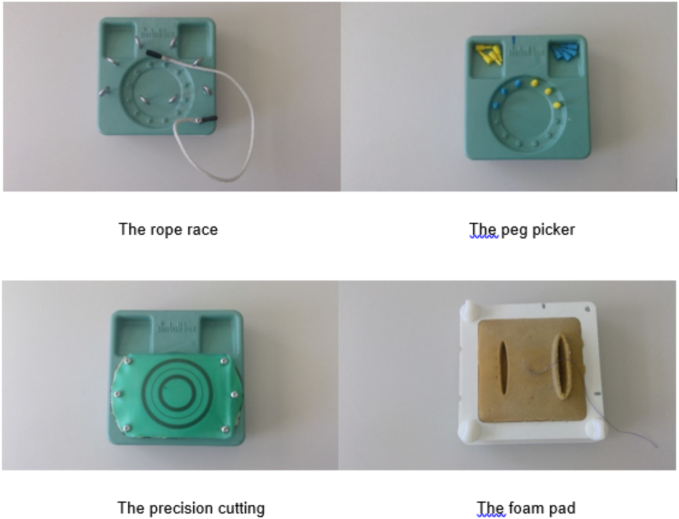
The specific tasks.

In the rope race task (Fig. 2), a thread had to be passed through several loops arranged in a circle in a specific direction. This exercise was repeated 10 times in each round. In the pin-picking task, the participants had to move several pins from one side of a device to the opposite side. This task was also repeated 10 times in each round.

In the precision cutting task, a round mark imitating a tissue sample was to be cut out in a circle as accurately as possible and picked up in such a way that the underlying material was not damaged. Each round was repeated 5 times. In the last exercise, a continuous suture had to be performed on a foam pad in the same way as in the exercise with the single knot [6].

### The simulator

**Fig. 3:**
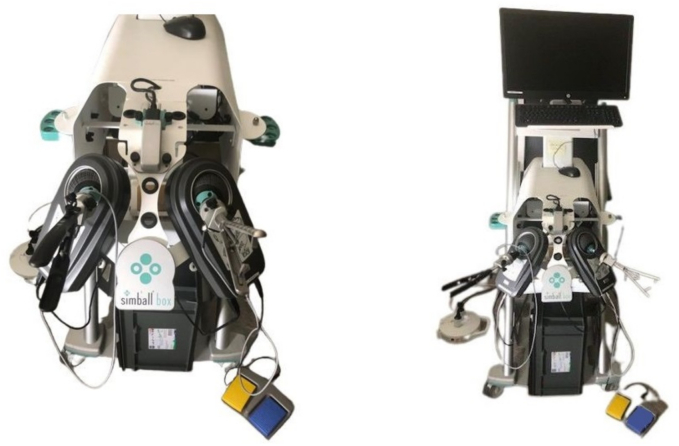
Simball® Box and installed Simball® Box simulator.

Simball® Box is a box trainer developed by G-Coder Systems from Västra, Sweden (now Surgicalscience) [7]. The simulator works with real surgical instruments equipped with specific motion sensors to record the specific movements and perform data quantification using metrical values. The surgical instruments are placed in an instrument holder connected to a ball joint. The instruments are fixed in the instrument holder to eliminate the axial movement of the instrument itself [1,6] (Fig. 3).

**Fig. 4:**
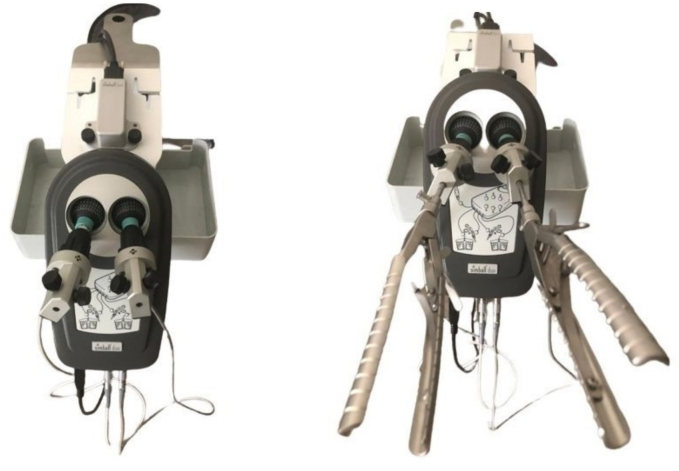
Simball® Duo simulator.

The simulator can recognize movements in 3 different axes based on the surface pattern on the ball joint. It can also analyse the depth of the instrument through the instrument holder, resulting in a 4D analysis of the instrument in the surgical field. The technology used is based on the above-mentioned surface pattern and makes it possible to analyse the exact position of the instrument in the box at a given time. At the same time, this measurement is updated hundreds of times per second, resulting in a detailed metric quantification and evaluation of the performance. Our laptop was operated with a Windows operating system [1,6]. Among other things, it can be used as a training simulator for uniportal VATS procedures [4] (Fig. 4).

### Curriculum questionnaire

The subjective opinion of the curriculum program was assessed using a standardized questionnaire filled out by the participants before the training to evaluate their previous experience with endoscopic surgery. At the end of the program, feedback on the training was obtained using the second part of the same questionnaire [6] (Annex 1).

### The statistical analysis of data

The data collected was stored directly in a Microsoft® Excel spreadsheet. These included the number of trials, linear distance, average speed, average acceleration, angular distance, uniformity of movement and time taken to complete a task [1,6]. The values were recorded in chronological order. Data were extracted and statistical analysis was performed using IBM SPSS Statistics software version 25.0. Values of *p* ≤ 0.05 were considered statistically significant. Quantitative variables were expressed as mean values (SD). The comparison between the three time points of simple knot tying before (T1), in the middle (T2), and after training (T3) was analyzed using one-way repeated measures analysis of variance (ANOVA) with “time” as a within-subject factor. Post hoc comparisons were performed using the least significant difference (LSD) method. The partial eta squared (ηp2) was used as a measure of effect size.

## Results

The study involved primarily 19 participants. Among them, were 15 medical students with no prior or limited surgical endoscopic skills and 4 surgical residents with previous exposure to surgical endoscopic procedures. Sixteen (16) participants successfully completed the curriculum (13 students, 3 residents) in the requested setting, and their data were included in the analysis; the data of one resident and two medical students were excluded because they did not complete all the exercises or the training in the designated form.

Of all recruited course participants, 10 were male and 9 were female (students: 9 female, 6 male; residents: 4 male). Of the 16 students recruited, all were in their 4th to 6th year of medical school. Of the participants who successfully completed the curriculum, 15 were right-handed and only one resident was left-handed dominant. The median age of the students was 25 years (range 24–30 years). Median age of the residents was 28 years (range 26–29).

Before the exercises began, a questionnaire was completed in which previous experience with endoscopic procedures was recorded. Of all the medical students, only one stated previous experience in endoscopic procedures (three years in total). Of the three participating surgical residents, one reported having <1 year of experience in laparoscopic surgery or video-assisted thoracic surgery (VATS). Two surgical residents had previous experience with either laparoscopic, multiportal or uniportal VATS procedures.

### Metrics and quantification

All metrics are presented detailed in Table 2.

**Table 2:** All metrics.

Diagramm all metrics	T1	T2	T3	*p*-Value	Comparison of T2 to T1 [%]	Difference of T2 to T1 [%]	Comparison of T3 to T1 [%]	Difference of T3 to T1 [%]	Comparison of T3 to T2 [%]	Difference of T3 to T2 [%]
Total time (sec)	707.5	223.57	144.77	<0.001	31.60	68.40	20.46	79.54	64.75	35.25
Distance overall (cm)	2227.39	837.97	568.3	<0.001	37.62	62.38	25.51	74.49	67.82	32.18
Average speed overall (mm/s)	16.27	19.35	20.09	<0.001	118.93	−18.93	123.48	−23.48	103.82	−3.82
Average speed time (mm/s) R	17.8	21.28	21.23	0.001	119.55	−19.55	119.27	−19.27	99.77	0.23
Average speed time (mm/s) L	14.31	18.15	19.19	<0.001	126.83	−26.83	134.10	−34.10	105.73	−5.73
Average acceleration overall (mm/s^2^)	752.77	919.18	903.68	0.041	122.11	−22.11	120.05	−20.05	98.31	1.69
Average acceleration (mm/s^2^) R	824.66	986.86	955.27	0.081	119.67	−19.67	115.84	−15.84	96.80	3.20
Average acceleration (mm/s^2^) L	680.89	835.48	852.1	0.036	122.70	−22.70	125.15	−25.15	101.99	−1.99
Motion smoothness overall (m/s^2^)	50.31	59.82	60.59	0.02	118.90	−18.90	120.43	−20.43	101.29	−1.29
Motion smoothness (m/s^2^) R	55.37	64.51	64.12	0.052	116.51	−16.51	115.80	−15.80	99.40	0.60
Motion smoothness (m/s^2^) L	45.26	54.65	57.04	0.017	120.75	−20.75	126.03	−26.03	104.37	−4.37
Total angular distance overall (radians)	350.51	143.38	99.05	<0.001	40.91	59.09	28.26	71.74	69.08	30.92
Total angular distance (radians) R	213.4	86.34	58.31	<0.001	40.46	59.54	27.32	72.68	67.54	32.46
Total angular distance (radians) L	137.11	57.03	47.67	<0.001	41.59	58.41	34.77	65.23	83.59	16.41

#### Total time (sec)

**Fig. 5:**
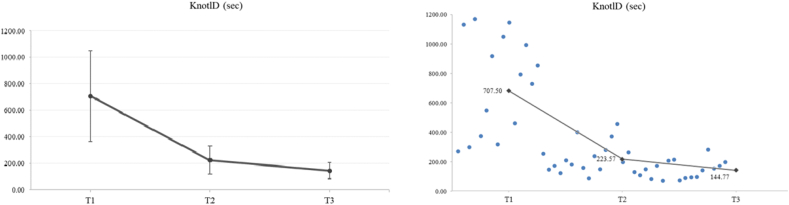
The results showed a significant main effect of “Time” for KnotlD (*F*_2,30_ = 39.70, *p* < 0.001, η_p_^2^ = 0.726). The mean values (SD) of KnotlD at T1, T2 and T3 (sec) were 707.5 (343.42), 223.57 (106.85), and 144.77 (61.04), respectively. In particular, the least significant difference (LSD) post hoc comparisons revealed a significant reduction of KnotlD in T2 compared to T1 (*p* < 0.001). A significant reduction was also observed in KnotlD in T3 compared to T2 (p < 0.001). Moreover, a significant reduction was observed in KnotlD in T3 compared to T1 (*p* < 0.001).

The mean time for completing the single-knot tie at T1, T2 and T3 (sec) were 707.5 (343.42), 223.57 (106.85), and 144.77 (61.04), presenting a significant improvement of **68,40 % from T2 to T1** (***p*** < **0.001**), an improvement of **35.25** **% from T3 to T2** (***p*** < **0.001**) and an overall improvement of **79.54** **% from T3 to T1** (***p*** < **0.001**) (Fig. 5).

#### Overall distance (cm)

**Fig. 6:**
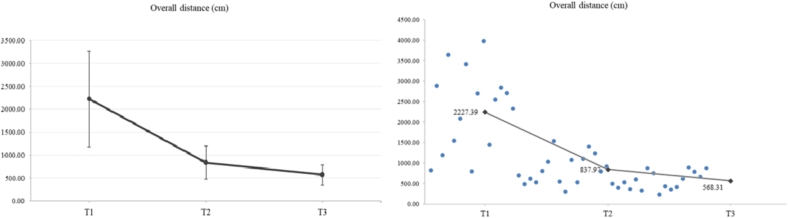
Concerning the overall distance (cm), a significant main effect was found (*F*_2,30_ = 34.19, *p* < 0.001, η_p_^2^ = 0.695). The mean values (SD) of overall distance at T1, T2 and T3 were 2227.39 (1046.17), 837.97 (363.37) and 568.31 (219.53), respectively. In particular, LSD post hoc comparisons revealed a significant reduction of overall distance in T2 compared to T1 (*p* < 0.001). A significant reduction was also observed in overall distance in T3 compared to T2 (*p* = 0.007). Moreover, a significant reduction was observed in overall distance in T3 compared to T1 (*p* < 0.001).

Concerning the overall distance (cm), a significant main effect was recorded. The mean values (SD) of overall distance at T1, T2 and T3 were 2227.39 (1046.17), 837.97 (363.37) and 568.31 (219.53), respectively. A significant improvement of **62.38** **% from T2 to T1** (***p*** < **0.001**), an improvement of **32.18** **% from T3 to T2** (***p*** < **0.007**) and an overall improvement of **74.49** **% from T3 to T1** (***p*** < **0.001**) was recorded (Fig. 6).

#### Average speed (mm/s)

**Fig. 7:**
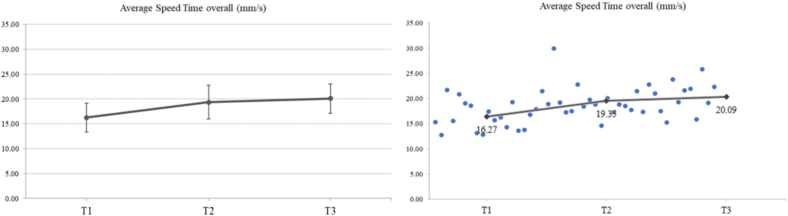
A significant increase for average speed (mm/s) was recorded (*F*_2,30_ = 13.68, *p* < 0.001, η_p_^2^ = 0.477). The mean values (SD) of average speed at T1, T2 and T3 were 16.27 (2.9), 19.35 (3.41) and 20.09 (2.96), respectively. In particular, LSD post-hoc comparisons revealed a significant increase in average speed at T2 compared to T1 (*p* = 0.002). Moreover, a significant increase was also observed in average speed at T3 compared to T1 (p < 0.001).

A significant increase was observed in the overall average speed (mm/s). The mean (SD) values of average speed at T1, T2 and T3 were 16.27 (2.9), 19.35 (3.41) and 20.09 (2.96), respectively, presenting an increase of **18.93** **% from T2 to T1** (***p*** < **0.002**) and an increase of **23,48 % from T3 to T1** (***p*** < **0.001**). No significant difference was found **from T3 to T2** (Fig. 7).

For the right hand, a significant increase in average speed of **19,55 % was found at T2 compared to T1** (***p*** = **0.001**) and an increase in average speed **19,27 % was found at T3 compared to T1** (***p*** = **0.003**). For the left hand, the values showed a significant increase in average speed at T2 compared to T1 by **26.83** **%** (***p*** = **0.002**) and a significant increase in average speed at T3 compared to T1 by **34.10** **%** (***p*** < **0.001**). There were no significant differences in average speed at T3 compared to T2 for both hands. Detailed data are shown in Table 2.

#### Average acceleration (mm/s^2^)

**Fig. 8:**
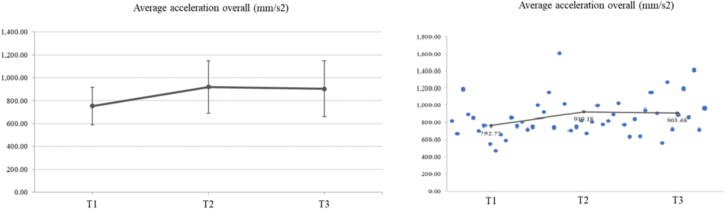
The repeated ANOVA results showed a significant increase for the tree time points on average acceleration (F2,30 = 3.57, p = 0.041, ηp2 = 0.192). The means (SD) of average acceleration (mm/s2) at T1, T2, and T3 were 752.77 (163.96), 919.18 (228.11) and 903.68 (244.28), respectively. LSD post-hoc comparisons showed a significant increase in acceleration at T2 compared to T1 (p = 0.007). No other significant difference was detected.

The results showed a significant increase in mean acceleration (mm/s^2^) for each hand individually and overall. The mean values of the average acceleration at T1, T2 and T3 were 824.66 (147.11), 986.86 (240.53) and 955.27 (269.78) for the right hand and 680.89 (208.25), 835.48 (236.4) and 852.1 (239.15) for the left hand, revealing an increase of **19.67** **% for the right hand** (***p*** = **0.015**) and **22.70** **% for the left hand** (***p*** = **0.014**) **at T2 compared to T1**, respectively. Moreover, there was an increase in mean acceleration of 25.15 **% for the left hand at T3 compared to T1** (***p*** = **0.041**). In addition, the mean values of total acceleration at T1, T2 and T3 were 752.77 (163.96), 919.18 (228.11) and 903.68 (244.28), respectively. At T2, a significant increase in acceleration of 22.11 % was observed compared to T1 (*p* = 0.007). No other significant difference was found (Fig. 8).

#### Angular distance (radians)

**Fig. 9:**
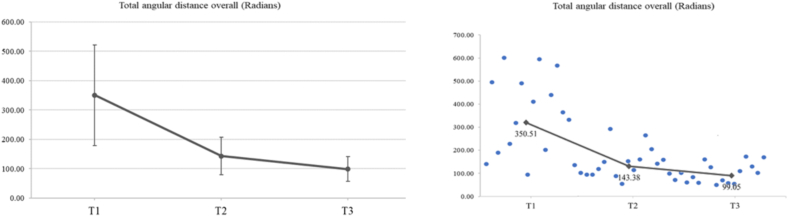
A significant decrease for total angular distance was found (*F*_1.19,17.91_ = 28.78, *p* < 0.001, η_p_^2^ = 0.657). The mean values (SD) of total angular distance (Radians) at T1, T2 and T3 were 350.51 (171.46), 143.38 (64.43) and 99.05 (42.48), respectively. Specifically, LSD post-hoc comparisons revealed a significant decrease in total angular distance at T2 (p < 0.001) and T3 (p < 0.001) compared to T1. In addition, a significant reduction was also found in total angular distance at T3 compared to T2 (*p* = 0.010).

The angular distance in radians (yaw, pitch and roll movement) was measured for each hand and overall. For the right hand, a significant reduction of 59.54 % at T2 compared to T1 (*p* < 0.001), a reduction of 32.46 % from T3 compared to T2 (*p* = 0.003) and of 72.68 % at T3 compared to T1 (*p* < 0.001) was revealed. For the left hand, a significant reduction of 58.41 % at T2 compared to T1 (*p* = 0.001) and of 65.23 % at T3 compared to T1 (p < 0.001) was documented. Overall, the total angular distance in radians was reduced by 59.09 % at T2 compared to T1 (*p* < 0.001), by 30.92 % at T3 compared to T2 (*p* = 0.010) and by 71.74 % at T3 compared to T1 (p < 0.001) (Fig. 9).

#### Motion smoothness (m/s^3^)

**Fig. 10:**
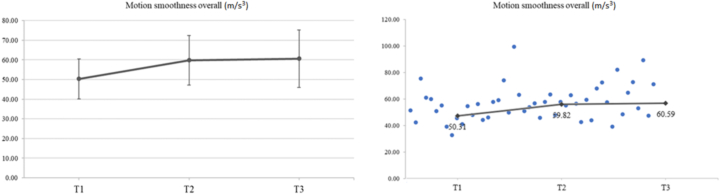
Regarding the motion smoothness (m/s^3^), a significant increase was recorded (*F*_2,30_ = 4.47, *p* = 0.020, *η*_*p*_^2^ = 0.229). The mean values (SD) of motion smoothness at T1, T2 and T3 were 50.31 (10.25), 59.82 (12.64) and 60.59 (14.66), respectively. LSD post-hoc comparisons showed a significant increase in motion smoothness at T2 compared to T1 (*p* = 0.008). A significant increase was also observed in motion smoothness at T3 compared to T1 (*p* = 0.031).

The smoothness of movement (m/s^3^) was also documented. For the right hand, a significant increase in movement smoothness of 16.51 % at T2 compared to T1 was documented (*p* = 0.017). For the left hand, an increase of 20.75 % at T2 compared to T1 (*p* = 0.012) and 26.03 % at T3 compared to T1 (*p* = 0.021) was found. Overall, an increase of 18.90 % at T2 compared to T1 (*p* = 0.008) and of 20.43 % at T3 compared to T1 (*p* = 0.031) (total) was measured (Fig. 10).

### Evaluation of the training curriculum

After the training, the sixteen participants who had successfully completed the curriculum were asked to evaluate the training curriculum and its potential benefits using a questionnaire. The positive feedback was recorded. In addition, the individual, subjective opinion of each participant was surveyed. Here, the residents generally stated that the beginning was hard, but that the improvement in their basic surgical skills was impressive and that they had benefited from the training. Some comments mentioned that technical parts of the study, such as the teaching videos at the beginning, the grip of the surgical clamp, or the surgical thread, should be improved for future studies, and the trainees stated that such a training curriculum should be a standard in surgical training.

## Discussion

### Simulator training as part of educational curricula

Alvarez Martinez et al. reported on the training program at their university hospital, in which all relevant minimally-invasive departments were involved. They demonstrated that the combined training led to efficient training and that a reduction in costs reduction was also observed due to the synergies utilized [7]. This speaks for the feasibility of structured simulator training in surgical specialties. The aviation industry can serve as a prime example of the successful integration of simulator training [8].

For the continuous training of endoscopic skills, the use of cadavers or synthetic models does not appear to be feasible. For this reason, simulator-based training is an essential part of training, according to Imaizumi et al. They conducted a one-year continuous prospective, observational study with five surgical residents in different stages of surgical training. The participants repeated specific primary tasks in minimally invasive surgery. In summary, their results showed an improvement in task completion time for all participants. They conclude that continuous simulator training can play a role in surgical skills training [10].

The pilot training curriculum, where simulator training has become an integral part of training, could provide us with the knowledge to demonstrate a similar success story in surgical training curricula.

The present study showed that the participants were able to improve their basic endoscopic skills. Regarding linear distance, total time, average speed and total angular distance, the mean values between initial knot tying before training and knot tying after training showed a significant increase in skills. For linear distance, the reduction in total distance between the knot tying mid- and post-training did not reach statistical significance (*p* = 0.007). This finding can be interpreted as this specific parameter decreases faster at the beginning and individual learning of the participant can be observed after the initial learning on the simulator. In the median, however, learning is concentrated on the first round of exercises. For the average acceleration, an initial significant reduction was observed from before to halfway through the training. The parameters examined show that the learning curve rises most steeply between the node before and in the middle of training. However, our data do not provide sufficient evidence that a training period of 8 h, e.g. one day on a simulator, is sufficient to obtain the first primary endoscopic skills. Simulator training can complement a more skills-based approach to surgical training. The benefits of simulator training have been mentioned many times and are numerous and understandable. The next generation of surgeons needs time and cost-effective training tools with a greater focus on patient safety. Assessment or standardized simulator training will be incorporated into the resident curriculum. Our observations correlate with support for the implementation of a structured training curriculum. Progress in basic endoscopic surgical skills is possible through repetitive training, and it seems feasible to master the initial phase of the learning curve with simulator training. The objective parameter showed a significant increase in performance in the study participants, which was supported by subjective satisfaction with the endoscopic training. At Inselspital Bern, Department of Thoracic Surgery, we will integrate this form of training into the structured surgical residency program.

## Conclusion

It appears that simulator training has the momentum and expected efficiency to be an important part of future surgical training curricula. By training on an endoscopic simulator, trainees can quickly learn basic endoscopic skills and progress through the initial phase of the learning curve in a safe and efficient atmosphere (dry or wet lab).

While there are several simulators with haptic or virtual feedback that allow quantification of training outcomes, there are no objective parameters to date that describe the point at which the most effective outcomes or skills can be achieved. Further research should be devoted to this objective and data analysis [4,9].

In this project, participants included students with no previous exposure or surgical experience. The quantified results showed that no prior experience was required for entry into such a curriculum.

We strongly advocate and recommend the implementation of our simulator training curriculum in all surgical facilities where endoscopic skills are required for minimally invasive procedures.

## Ethical approval

Ethical review and approval were waived for this study, as no patients or patients' data were involved.

## Funding sources

This research received no external funding.

## CRediT authorship contribution statement

**Marc Fischer:** Writing – original draft, Resources, Project administration, Investigation, Data curation. **Michail Galanis:** Writing – review & editing, Writing – original draft, Supervision, Methodology, Formal analysis, Data curation. **Konstantinos Gioutsos:** Writing – review & editing, Validation. **Jon Andri Lutz:** Methodology, Conceptualization. **Filipe Azenha Figueiredo:** Methodology, Conceptualization. **Patrick Dorn:** Writing – review & editing, Validation, Supervision, Project administration.

## Declaration of competing interest

The authors declare no conflict of interest.

## References

[1] Hagelsteen K., Sevonius D., Bergenfelz A., Ekelund M. (2016). Simball box for laparoscopic training with advanced 4D motion analysis of skills. Surg Innov.

[2] Papanikolaou I.G., Haidopoulos D., Paschopoulos M., Chatzipapas I., Loutradis D., Vlahos N.F. (2019). Changing the way we train surgeons in the 21th century: a narrative comparative review focused on box trainers and virtual reality simulators. Eur J Obstet Gynecol Reprod Biol.

[3] FMH-Ärztestatistik, Schweiz.

[4] Rodriguez-Paz J.M., Kennedy M., Salas E. (2009). Beyond “see one, do one, teach one”: toward a different training paradigm. Postgrad Med J.

[5] Brodmann Maeder M. (2023). Meine erste Zeit am SIWF. Schweiz Ärzteztag [Internet]. https://doi.emh.ch/saez.2023.21476.

[6] Fischer (2022).

[7] Simball Box – True metrics and haptics in box training [Internet]. Surgical Science. https://surgicalscience.com/simulators/simball-box/.

[8] Álvarez Martínez L., Ruiz Aja E., Valdivieso Castro M.P. (2022). Common surgical training program: standardization of learning quality. Programa de formación quirúrgica común: uniformidad en la calidad del aprendizaje. Cir Pediatr.

[9] Reznick R.K., MacRae H. (2006). Teaching surgical skills—changes in the wind. N Engl J Med.

[10] Imaizumi K., Ichikawa N., Homma S. (2023). Effect of continuous box-trainer training on laparoscopic skills of surgical residents: a prospective, observational study. In Vivo.

